# Gene gun DNA immunization of cattle induces humoral and CD4 T-cell-mediated immune responses against the *Theileria parva* polymorphic immunodominant molecule

**DOI:** 10.1016/j.vaccine.2019.02.009

**Published:** 2019-03-14

**Authors:** Lindsay M. Fry, Reginaldo G. Bastos, Brad C. Stone, Laura B. Williams, Donald P. Knowles, Sean C. Murphy

**Affiliations:** a Animal Disease Research Unit, Agricultural Research Service, US Department of Agriculture, P.O. Box 646630, Pullman, WA 99164, USA; b Department of Veterinary Microbiology & Pathology, Washington State University, P.O. Box 647040, Pullman, WA 99164, USA; c Department of Laboratory Medicine, University of Washington Medical Center, 1959 NE Pacific St., Seattle, WA 98195, USA; d Department of Microbiology, University of Washington, 750 Republican St., Box 358070, Seattle, WA 98109, USA; e Center for Emerging and Re-emerging Infectious Diseases, 750 Republican St., Seattle, WA 98109, USA

**Keywords:** BAEC, bovine aortic endothelial cells, BoLA, bovine leukocyte antigen, CBC, complete blood count, CO, codon-optimized, ECF, east coast fever, ELISA, enzyme linked immunosorbent assay, GG, gene gun, HEK, human embryonic kidney cells, IFNγ, interferon gamma, ITM, infection and treatment method, MHC, major histocompatibility complex, PIM, polymorphic immunodominant molecule, DNA vaccination, East Coast Fever, Gene gun immunization, Cattle, Vaccine

## Abstract

*Theileria parva* kills over one million cattle annually in sub-Saharan Africa. Parasite genetic complexity, cellular response immunodominance, and bovine MHC diversity have precluded traditional vaccine development. One potential solution is gene gun (GG) immunization, which enables simultaneous administration of one or more DNA-encoded antigens. Although promising in murine, porcine, and human vaccination trials, bovine GG immunization studies are limited. We utilized the model *T. parva* antigen, polymorphic immunodominant molecule (PIM) to test bovine GG immunization. GG immunization using a mammalian codon optimized PIM sequence elicited significant anti-PIM antibody and cell-mediated responses in 7/8 steers, but there was no difference between immunized and control animals following *T. parva* challenge. The results suggest immunization with PIM, as delivered here, is insufficient to protect cattle from *T. parva*. Nonetheless, the robust immune responses elicited against this model antigen suggest GG immunization is a promising vaccine platform for *T. parva* and other bovine pathogens.

## 1 Introduction

The tick-borne, apicomplexan parasite *Theileria parva* kills over one million cattle annually in sub-Saharan Africa [1]. Infection results in a clinical syndrome known as East Coast Fever (ECF), characterized by pyrexia, lymphadenopathy, and respiratory failure [2]. Mortality rates are highest in European cattle breeds imported for higher meat and milk yields, and most losses are incurred by smallholder pastoralist farmers [3]. Improved control of *T. parva* via next-generation vaccine development is a critical aspect of international aid programs to combat poverty and starvation in sub-Saharan Africa.

Immune responses to *T. parva* consist of sporozoite-specific antibody responses and major histocompatibility complex (MHC) class I- and II-restricted T cell responses to schizont-infected lymphocytes [4], [5], [6], [7]. Protective immunity is elicited by sub-lethal natural infection and by the infection and treatment method (ITM), whereby cattle are infected with cryopreserved, *T. parva*-infected tick stabilate and co-treated with oxytetracycline [1]. ITM effectively premunizes cattle against homologous strains of *T. parva* and is integral to ECF prevention in some areas. Unfortunately, widespread adoption of ITM throughout sub-Saharan Africa has been severely limited by production and implementation costs and liquid nitrogen storage requirements. Concerns regarding the induction of a *T. parva* carrier state in ITM-immunized animals and limitations of cross-strain protection have further constrained ITM use [1], [8], [9]. Longer term ECF control strategies include ITM improvement and next-generation vaccine development.

To date, several *T. parva* vaccine trials utilizing various antigens and delivery platforms have been conducted [10], [11], [12]. In some trials, a proportion of immunized animals developed *T. parva*-specific immune responses, and a subset of these animals were protected from lethal challenge [10], [11], [12]. Although promising, the marginal success of these trials highlights challenges inherent in subunit vaccine development for complex intracellular pathogens of outbred host species. Indeed, genetic complexity [13] and strain variation of *T. parva* [14], [15], [16], coupled with immunodominance of the protective cellular immune response [15], [17] and diverse nature of bovine MHC loci [18], [19], [20], [21] have precluded development of a traditional *T. parva* subunit vaccine with population-wide efficacy.

A potential solution to these challenges is gene gun (GG) immunization, also known as particle-mediated epidermal delivery DNA immunization, which enables simultaneous intradermal inoculation of one or more DNA-encoded antigens [22], [23], [24], [25]. DNA-encoded antigens are biolistically delivered into epidermal and dermal professional and non-professional antigen presenting cells (APC) where they are expressed, processed, and elicit an immune response [22]. Due to direct deposition of DNA-encoded antigens in the nucleus of dermal APCs, GG DNA immunization requires 10 to 100-fold less DNA than conventional intramuscular DNA immunization yet elicits much stronger humoral and cell-mediated immune responses [26]. Additionally, the flexible nature of the platform allows inclusion of diverse DNA-encoded, co-stimulatory molecules and genetic adjuvants to enhance immune response development.

GG DNA immunization has been successfully utilized in vaccination trials for viral and neoplastic diseases in humans, primates, pigs and mice, and was recently used in mice to discover new *Plasmodium yoelii* sporozoite candidate antigens [22], [25], [27], [28]. To our knowledge, GG DNA immunization using complex protozoal antigens has never been tested in a large, outbred species such as cattle. Here, we assessed bovine GG DNA immunization using the *T. parva* polymorphic immunodominant molecule (PIM) antigen in Holstein steers, and made modifications to increase PIM immunogenicity. PIM is comprised of a central variable region flanked by conserved N- and C-terminal ends, ranges in size (based on strain) from 62 kDa, and is a good model of structurally-complex protozoal proteins [29]. PIM is highly expressed by both the sporozoite and schizont stages [30] and plays a role in sporozoite lymphocyte entry during early infection [31]. As its name implies, PIM is the target of vigorous immune responses during *T. parva* infection [32]. Due to the universality of the robust antibody response to PIM during *T. parva* infection, recombinant PIM is used in the *T. parva* diagnostic ELISA test [33]. Since this ELISA is well-characterized and validated, it can be used to demonstrate the development of a humoral immune response to GG DNA immunization using PIM as a model. Although widely used in *T. parva* diagnostic assays, the protective potential of PIM as a vaccine antigen had not been formally assessed in a large group of cattle until the present study. Our study provides a potential path for using GG DNA immunization and codon optimization to increase the immunogenicity of *T. parva* vaccine candidates to achieve high rates of protection against challenge.

## 2 Materials and methods

### 2.1 Cattle

All animal experiments were approved by the Washington State University Institutional Animal Care and Use Committee (protocol #4980). Therapeutic drugs were administered according to manufacturers’ instructions. This study utilized ten, 3–12 month-old, MHC class I A10 or A14 heterozygous haplotype-matched [34] Holstein steers (Table 1) from dairies in central Washington. Cattle were quarantined at the USDA-ARS Animal Disease Research Unit (ADRU) research barns for two weeks before the start of the study and received regular health checks from licensed veterinarians. After quarantine, calves were housed in small groups at the USDA-ARS-ADRU disease research barn. Pre-immunization and pre-challenge complete blood counts (CBCs) and serum chemistry panels were normal, and all calves tested negative on a pre-immunization *T. parva* PIM enzyme-linked immunosorbent assay (ELISA) (data not shown).

**Table 1:** MHC class I and class II genotypes of immunized and control steers.

Steer	MHC I	MHC II
489	A10/A12	1001/1501
1424	A10/A15	1001/0201
780	A10/A11	1001/0101
901	A10/A19	1001/0901
148	A14/A15	0902/1101
790	A14/A11	0101/1401
807	A14/A083	0902/0101
817	A14/A084	0902/1401
141*	A10/A11	1001/0101
1413*	A14/A11	0202/0701

* Control steer.

### 2.2 Cloning of expression constructs

All constructs were cloned into NTC9384R UbA76 antibiotic free vector (NatureTechnologies Inc., Lincoln, NE). The vector backbone was amplified using the primers (5′-gagagagtcgacggtggcttctcgacgacggtttgt-3′ and 5′-cccggggagatctttttccctctgccaaaaattatggg-3′) to delete the Ubiquitin tag and the product was *Dpn*I digested. The native *T. parva* Muguga PIM coding sequence (GenBank Accession number AAGK01000004 [35]) was amplified from genomic DNA using PIM-specific primers (5′-agagagtcgacatgaagatctttccctttttatttatatttccatttttattaaaatta-3′ and 5′-gagagacccgggttaacaacaatcttcgttaatgcgagaaaaagagttgc-3′). Vector and insert were digested with *Sal*I and *Xma*I, gel purified, ligated and transformed into chemically-competent NTC821601 *E. coli*. Clones were isolated and sequence verified. The codon-optimized (CO) PIM coding sequence was designed (Supplementary Fig. 1), commercially synthesized (GeneWiz, Plainfield, NJ USA), and subcloned into the NTC vector using *Sal*I and *Xma*I in a similar manner. Plasmids were grown in Luria broth (LB) and purified using an Endo-free Maxi Kit according to the manufacturer’s instructions (Qiagen, Hilden, Germany). A construct encoding the genetic adjuvant *E. coli* heat-labile enterotoxin (LT) was obtained from Debora Fuller (University of Washington, Seattle, WA) and prepared in a similar manner.

### 2.3 Assessment of *in vitro* PIM expression

To evaluate *in vitro* expression of PIM, human embryonic kidney (HEK) 293 t cells (ATCC# CRL-11268) and bovine aortic endothelial cells (BAEC) (Sigma-Aldrich, St. Louis, MO) were transiently transfected with NTC-PIM native or NTC-PIM-CO using PEI transfection reagent (Sigma-Aldrich). Briefly, cells were seeded at 2 × 10^5^ cells/well in six-well plates and incubated overnight at 37 °C and 5% CO_2_. Cells were next incubated with a transfection mix containing 2 µg plasmid DNA and PEI in OPTI-MEM (Gibco, Gaithersburg, MD). After incubation for 4 h, the transfection mix was replaced by DMEM (Gibco) with 10% FBS and cells were collected at 24 or 48 h post-transfection into Cell Culture Lysis Reagent (Promega, Madison, WI). pMaxGFP (Lonza, Basal, Switzerland) was used as a positive control for the transfections.

PIM protein expression in cell lysates was assessed by immunoblotting using standard methods [36]. Detection of PIM was achieved via incubation for one hour at room temperature with anti-PIM monoclonal antibody (mAb) clone ILS40.2 (1 µg/mL) [30]. Membranes were then washed in PBS-T and incubated for 1 h at room temperature with anti-mouse HRP (1:2500) (Sera Care, Milford, MA). Immune complexes were visualized using an enhanced chemiluminescence method (Amersham ECL; GE Healthcare, Pittsburg, PA). AlphaView® SA software (ProteinSimple) was used to perform densitometry analysis of immunoblot protein bands to measure the relative expression of PIM in HEK 293 t and BAEC cells.

### 2.4 Gene gun cartridge loading

Cartridges were loaded using traditional methods [28]. Cartridge batches were quality controlled by elution of single cartridges with 50 µL of water and quantified for DNA concentration using a NanoDrop (Thermo Scientific, Waltham, MA). Typical DNA yield per cartridge was 0.5 µg.

### 2.5 Gene gun immunization of cattle

A Helios Gene Gun (Bio Rad, Hercules, CA) was used for GG DNA delivery. Eight calves received four inoculations with pNTC-PIM-native (native sequence PIM) followed by immunogenicity evaluation using PIM ELISA. Due to the absence of consistent immune response development following pNTC-PIM native immunization, cattle were subsequently inoculated four times with pNTC-PIM-CO (codon-optimized PIM). Two calves were inoculated eight times with empty NTC plasmid and were used as controls. Inoculations were separated by 45–60 days. Before each inoculation, an approximately 15 cm^2^ area on the left side of the neck was closely shaved and cleaned with 70% ethanol. Once the skin was prepared, ten cartridges were administered at separate foci within the shaved area using 500 psi helium. Thus, over the course of the entire experiment, immunized cattle were inoculated with 40 pNTC-PIM-native cartridges (20 µg DNA) and 40 pNTC-PIM-CO cartridges (20 µg DNA), and control cattle were inoculated with 40 µg pNTC control plasmid. Peripheral blood was collected from each animal at various time points to assess the immune response.

### 2.6 Humoral immune response in PIM gene gun-immunized cattle

Serum samples were tested for the presence of anti-PIM antibodies by ELISA (ILRI, Nairobi, Kenya) per kit instructions [33] using positive and negative control sera included in the kit. This assay utilizes *E. coli* –expressed, full-length recombinant PIM antigen [33]. Plates were read at 405 nm (OD_405_) using a Multiskan MCC ELISA reader (Thermo-Fisher). The assay was performed in duplicate for each animal at each time point. Samples were considered positive if the observed OD_405_ was greater than or equal to three standard deviations above the OD_405_ of negative control animals.

To determine the predominant antibody isotype(s) comprising the humoral immune response to PIM immunization, the PIM ELISA was modified to use HRP-labeled anti-bovine IgG1 or similarly-labeled IgG2 secondary antibodies (both from Invitrogen and used at 1:500).

Immunoblotting was used to verify recognition of full-length, eukaryotic-cell-expressed codon-optimized PIM by immunized calf sera as described above. After blocking, membranes were incubated for one hour at room temperature in sera from immunized cattle diluted 1:2 or in ILS40.2 anti-PIM monoclonal antibody as described above. To reduce background, bovine serum samples were adsorbed with HEK293 cell lysate for 48 h at 4 °C prior to incubation with the membrane. After washing, membranes were incubated for one hour at room temperature with anti-bovine HRP (1:2500, SeraCare) or anti-mouse HRP (1:2,500 SeraCare), and complexes revealed using ECL as described above.

### 2.7 *T. parva* infected cell lysates

*T. parva* infected lymphocyte cell lines were established and maintained using standard methods [37]. Lysates of *T. parva*-infected cell lines and uninfected PBMC were used as antigen to assess cellular immune response development following PIM immunization. Briefly, 3 mL of *T. parva*-infected cell culture (3 × 10^6^ cells/mL) with a 40–60% infection prevalence, was lysed via two cycles of freezing (−80 °C) and thawing. Lysate was centrifuged twice for 3 min at 10,000*g*, supernatant discarded, and the pellet re-suspended in 400 µL of complete RPMI. Lysate was then tested by ELISpot assay (described below) to verify that it lacked soluble IFNγ (data not shown), and stored at −80 °C until use in immunological assays.

### 2.8 IFNγ response of PIM gene gun immunized cattle

One week after the final PIM inoculation, PBMCs from immunized and control steers were isolated from whole blood using density centrifugation with Histopaque (Sigma-Aldrich) to assess the *ex vivo T. parva*-specific immune response following PIM immunization. Production and secretion of IFNγ by PBMC stimulated overnight with 20 µL *T. parva*-infected cell lysate was measured using ELISpot assays (MabTech, Cincinnati, OH) per the manufacturer’s instructions. As a positive control for IFNγ production, PBMC from each animal were exposed to 20 ng/mL phorbol 12-myristate 13-acetate (PMA) (Sigma) plus 1 µg/mL ionomycin (Sigma), and PBMCs incubated without antigen served as negative controls. All cell cultures were performed in triplicate. Plates were read and analyzed using an Immunospot ELISpot reader (Cellular Technology Limited, Shaker Heights, OH). For each steer, the mean number of spot forming units (SFU) generated after PBMC incubation with *T. parva* infected cell lysate was compared to the mean number of SFU generated after incubation of PBMC alone using a two-tailed student’s T-test (α < 0.05).

### 2.9 *T. parva* challenge

Three weeks after the last immunization, cattle were challenged via subcutaneous inoculation of 1 mL cryopreserved Pullman 2015/4 *T. parva* Muguga sporozoite stabilate. Beginning three days post-challenge, animals were physically examined daily, and rectal temperature, pulse and respiratory rate recorded. Beginning seven days post-challenge, CBCs were assessed daily, and serum chemistry panels assessed weekly. Cattle that developed fever greater than 40.2 °C were treated with flunixin meglumine (MWI Animal Health, Boise, ID). Cattle that developed evidence of respiratory distress were euthanized via intravenous injection of pentobarbital (Fatal Plus, Vortech Pharmaceuticals, Dearborn, MI). A necropsy was performed on all cattle, and sections of lung and lymph node were collected and fixed in 10% neutral buffered formalin for histopathology and PIM immunohistochemistry, as previously described [2].

### 2.10 *T. parva* p104 qPCR

To determine the level of *T. parva* parasite density, quantitative PCR (qPCR) targeting the single-copy *T. parva* p104 gene was performed as previously described [38]. CFX Manager™ Software (Bio-Rad) was used to analyze the qPCR data. Samples were run in duplicate, with numbers of p104 copies presented as absolute numbers determined by the standard curve. Efficiency of amplification and melt curve analyses were performed to evaluate analytical sensitivity and specificity of the qPCR [2].

## 3 Results

### 3.1 Humoral response elicited by pNTC-PIM-native immunization

**Fig. 1:**
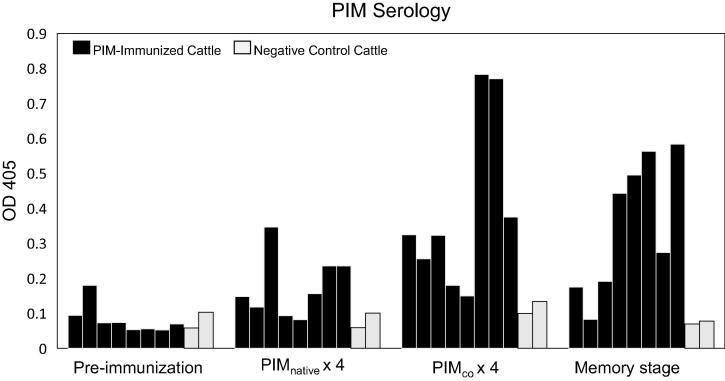
PIM serology results for immunized and control steers. PIM ELISAs were performed before immunization, one week after the final of four inoculations with the native PIM construct (PIM_native_ × 4), one week after the final of four inoculations with the mammalian cell codon-optimized (CO) PIM construct (PIM_CO_ × 4), and four months after the final inoculation (memory phase). The PIM ELISA uses *E. coli-*expressed, full-length *T. parva* Muguga PIM antigen. Black bars show individual immunized steers, grey bars show individual control steers. For each time point, bars represent the mean OD_405_ of duplicate wells. Responses were considered significant if the OD_405_ was greater than 3 standard deviations above the mean OD_405_ of the control cattle (indicated by *).

One month after the final of four GG DNA immunizations with pNTC-PIM-native, blood was collected from all cattle and serum assayed for the presence of an anti-PIM antibody response. At this point, only 3/8 cattle exhibited significant anti-PIM antibody responses compared to negative control cattle (Fig. 1).

### 3.2 Codon optimization and *in vitro* expression of PIM

Due to limited immunogenicity and variability of the *in vivo* responses above, we next assessed *in vitro* PIM expression in a bovine-derived cell line following transfection with the native PIM construct versus a CO-PIM construct to determine if codon optimization would increase PIM expression efficiency. Codon optimization increased the GC-content of the PIM sequence (Supplementary Fig. 2A). To investigate the effect of codon optimization on expression, native and codon-optimized sequences of PIM were cloned into the NTC9384R UbA76 plasmid as described above and used to transiently transfect HEK 293 t or BAEC cells.

Immunoblot analysis using the anti-PIM mAb ILS40.2 showed PIM expression in HEK 293 t cells was markedly increased in cells transfected with the CO sequence compared to the native sequence. Interestingly, BAEC were unable to efficiently express the native sequence, but expressed easily detectible levels of PIM when transfected with the CO sequence (Supplementary Fig. 2B). Densitometry revealed a greater than 2.5-fold increase in PIM expression in HEK 293 t cells transfected with plasmid containing the CO sequence compared to native sequence (Supplementary Fig. 2C).

Collectively, these data demonstrate a significant increase in *in vitro T. parva* PIM expression by transiently transfected when a mammalian CO sequence was used, especially in the bovine cell line.

### 3.3 Humoral response elicited by pNTC-CO-PIM immunization

**Fig. 2:**
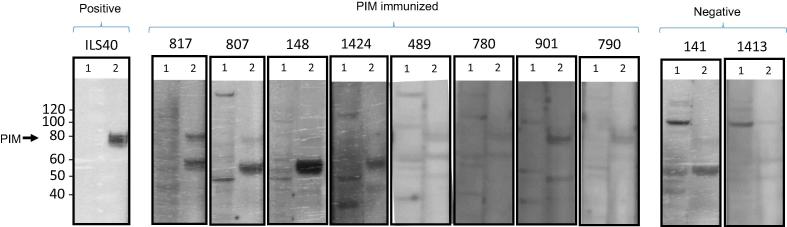
Immunoblot demonstrating recognition of HEK-cell expressed, codon-optimized (CO), intronless *T. parva* PIM by sera from gene gun-immunized steers. Animal identification numbers are indicated above each panel. For each panel, lane 1 contains control, non-transfected HEK 293 t cell lysate and lane 2 contains CO-PIM-transfected HEK 293 t cell lysate. Anti-PIM monoclonal antibody ILS40.2 was used as a positive control. The monoclonal antibody and sera from immunized cattle recognize a protein of approximately 77 kDa, consistent with PIM in lane 2 of each panel. This band is not present in lanes containing non-transfected HEK 293 t lysate or in panels from control steers.

Due to the poor expression profile of native PIM by bovine cells *in vitro* and the significant enhancement of expression when CO-PIM was used, we hypothesized that the native construct was poorly expressed *in vivo,* leading to reduced antigen production and resultant inconsistent antibody response. To test this hypothesis, we immunized all cattle an additional four times using the pNTC-PIM-CO construct. Two weeks after the last inoculation, 7/8 cattle exhibited significant anti-PIM antibody responses compared to the negative control cattle using the PIM ELISA (Fig. 1), and immunoblots using sera from immunized cattle verified recognition of the full-length, mammalian-cell-expressed, codon-optimized PIM antigen (Fig. 2). Serum was re-assessed in the PIM ELISA four months later to evaluate the longevity of the antibody response, and 7/8 cattle continued to show significant anti-PIM antibody responses. One steer lacked a significant antibody response in the final PIM ELISA but was seropositive in the previous assay, such that only 6/8 cattle maintained positive antibody titers four months following immunization (Fig. 1).

To better characterize the antibody response to PIM, anti-PIM antibody isotyping was performed on serum collected four months after the last inoculation. In 7/8 animals, the IgG repertoire contained significantly (p < 0.05) higher levels of IgG2 than IgG1 (Table 2).

**Table 2:** Pre-challenge anti-PIM total IgG/IgG1/IgG2, OD405 (±Standard deviation).

Animal ID	Total IgG	IgG1	IgG2
1424	0.2135 (±0.0205)	0.0445 (±0.0007)	0.2380 (±0.1400)^a^
489*	0.0775 (±0.0049)	0.0425 (±0.0021)	0.0590 (±0.0001)^a^
807	0.2465 (±0.0233)	0.0465 (±0.0021)	0.0725 (±0.0063)
817	0.2415 (±0.0021)	0.0430 (±0.0001)	0.2205 (±0.0233)^a^
901	0.2390 (±0.0000)	0.0460 (±0.0001)	0.0705 (±0.0021)^a^
780	0.4810 (±0.0268)	0.0445 (±0.0007)	0.0510 (±0.0001)^a^
790	0.5075 (±0.0318)	0.0445 (±0.0007)	0.4270 (±0.0593)^a^
148	0.1620 (±0.0042)	0.0420 (±0.0014)	0.0930 (±0.0028)^a^

a *p* < 0.05, *t* test comparing OD405 of IgG1 and IgG2.

* This animal failed to develop a significant, long-lasting anti-PIM antibody response.

### 3.4 Cellular immune response elicited by CO-PIM immunization

**Fig. 3:**
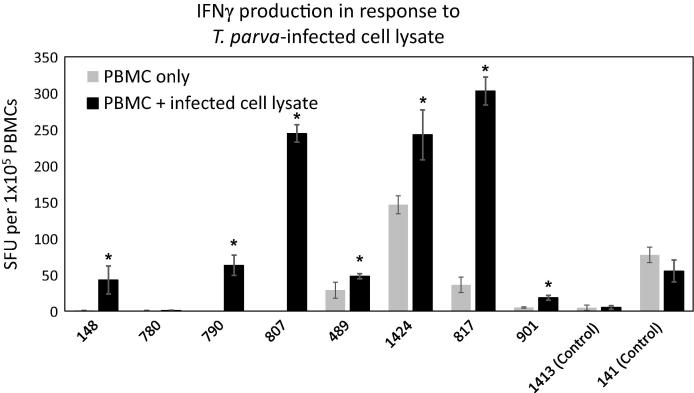
IFNγ cellular immune response to *T. parva*-infected cell lysate. An IFNγ ELISpot assay was performed 1 week after the final inoculation with codon-optimized PIM. The number of IFNγ spot forming units (SFU) are shown for each steer. Bars represent the mean SFU for triplicate wells. For each steer, the mean number of SFU generated after PBMC incubation with *T. parva*-infected cell lysate was compared to the mean number of SFU generated after incubation of PBMC alone using a two-tailed student’s T-test, *p < 0.05.

One week after the final inoculation with the pNTC-PIM-CO construct, blood was collected from all cattle and PBMCs used in IFNγ ELISpot assays to determine whether lymphocytes from PIM-immunized cattle would produce IFNγ in response to *T. parva-*infected cell lysate. Significant numbers of PBMC from 7/8 PIM-immunized cattle produced IFNγ in response to the *T. parva*-infected cell lysate compared to uninfected cell lysate, while PBMC from two control steers did not contain significant numbers of *T. parva*-specific, IFNγ-producing lymphocytes (Fig. 3). Since the steer in which an *ex vivo* cell-mediated immune response was not detected (Animal 780) developed a significant anti-PIM antibody response, and vice-versa (Animal 489), there was no apparent correlation between the development of cell-mediated and humoral immunity in these animals.

### 3.5 *T. parva* challenge

Upon challenge, there were no statistically significant differences in clinical responses to lethal *T. parva* challenge between the PIM-immunized and control cattle, and all cattle developed clinical signs (Supplementary Fig. 3) and gross and histologic lesions of severe ECF [2]. Histologically, the lungs and lymph nodes of all cattle contained abundant *T. parva* schizonts (Supplementary Fig. 4) [2].

## 4 Discussion

*T. parva* is the leading infectious cause of bovine mortality in sub-Saharan Africa [1], [3], [39]. Widespread adoption of ITM-immunization in the region is severely limited by its prohibitive cost, requirement for liquid nitrogen storage, necessity of antibiotic co-treatment, and induction of a *T. parva* carrier state in immunized cattle, resulting in possible ITM-strain transfer to new areas [1], [8]. Thus, in addition to ITM improvement, long-term ECF control strategies include development of a sustainable, cost-effective, next-generation vaccine [9]*.* Due to the complexity of the parasite genome and life cycle [13], the multifaceted nature of the protective *T. parva* immune response [15], and the marked genetic variability of outbred cattle populations [18], [19], [20], [21], killed organism vaccines and traditional, mono- or divalent subunit vaccines have not proven effective in ECF prevention [11]. It is likely that a protective vaccine will be multivalent -- comprised of antigens expressed by different parasite life stages and strains capable of eliciting vigorous humoral and cell-mediated immune responses in cattle of diverse genetic backgrounds.

For these reasons, GG DNA immunization is a promising *T. parva* vaccine platform. Not only does it enable inclusion of numerous antigens, it also allows addition of genetic adjuvants, including various immune-stimulating molecules, cytokines and shRNAs [23], [28]. Although widely tested and successful against several neoplasms and infectious organisms of mice and primates [22], [25], GG DNA immunization has only been used in a small number of herpesvirus vaccine trials in cattle [40], [41], [42]. In this study, we utilized the *T. parva* PIM antigen to optimize and test GG DNA immunization as a platform for vaccination against complex protozoal pathogens in cattle.

When GG DNA immunization was utilized to elicit protection against bovine herpesvirus-1 (BoHV-1), it was determined that mucosal delivery was superior to intradermal delivery [40]. Although mucosal delivery is an ideal route for respiratory pathogens like BoHV-1, intradermal delivery is the preferred route of immunization for hemoparasitic pathogens like *T. parva*, and if GG DNA immunization is to be used to prevent hemoparasitic infections, bovine immune responsiveness to intradermally delivered DNA-encoded antigens must be improved. In this study, we tested expression of native and CO-PIM *in vitro*, and followed induction of antibody responses when animals were serially vaccinated with native PIM and then with CO-PIM.

Expression of PIM by BAEC was undetectable when cells were transfected with the native PIM sequence, but was abundant when the CO-PIM construct was used for transfection. Similarly, when the native PIM construct was used for GG DNA immunization, only three cattle developed antibody responses. However, after receiving an additional four inoculations with the codon-optimized PIM construct, 7/8 cattle exhibited significant anti-PIM antibody responses. Since four of those animals failed to respond to PIM prior to inoculation with the CO construct, even after four inoculations, it is likely that the native construct was not expressed and processed to an extent sufficient to allow antigen binding and presentation in those steers, thereby abrogating the development of an immune response. These results indicate that CO greatly improves antigen expression and humoral immune response development to GG DNA immunization in cattle. We are currently investigating whether priming and boosting with CO-PIM alone can induce responses with greater efficiency.

Isotyping of the PIM-specific antibody response prior to *T. parva* challenge revealed that, in 7/8 animals, an IgG2 anti-PIM antibody response predominated. The role of different Ig isotypes in cattle has only been partially explored to date. Previous bovine studies suggests IgG2 is the major isotype responsible for classical pathway complement activation and is also important in opsonization [43]. Recently, a *T. parva* vaccine study using the antigen p67C demonstrated that significant anti-p67 IgG2 titers correlated positively with protection from challenge [12]. Numerous studies have shown that bovine IgG2 production is enhanced by IFNγ during Th1-skewed immune responses [44], [45], [46], [47], [48]. Due to the intracellular nature of *T. parva,* the development of a Th1 response is critical to host survival, and thus, must be elicited by a protective subunit vaccine.

In addition to the robust IgG2 response, 7/8 of the immunized cattle in our experiment also developed a significant Th1 T- cell response following GGDNA PIM immunization. Indeed, significant numbers of cells from these cattle produced IFNγ following overnight, *ex vivo* exposure to *T. parva-*infected cell lysate. Since the PIM antigen is an antibody target during natural infection [33], [49], in-depth analysis of the T-cell response was not performed in this study. However, the fact that responses in this group of immunized cattle were biased to a Th1, IFNγ and IgG2-predominant response suggests that our bovine GG DNA vaccine modifications, including mammalian cell codon-optimization, have greatly enhanced the utility of this vaccine platform for this complex livestock disease.

Despite the robust humoral and cell-mediated immune responses elicited by our PIM immunizations, there was no significant difference in challenge outcome between immunized and control steers in this study. This result was not unexpected, and its cause is likely multifactorial. First, immune-protection in *T. parva* is associated with the development of a CD8^+^ cytotoxic T-lymphocyte response, the antigenic target(s) of which are entirely dependent upon the MHC class I genotype and T-cell receptor repertoire of individual animals [4], [10], [17], [50], [51]. It is possible that the steers utilized in our experiments lacked the MHC class I background to efficiently bind and present PIM epitopes.

Second, although cattle almost always develop robust anti-PIM antibodies during natural infection, bovine anti-PIM antibodies have never demonstrated sporozoite neutralizing capabilities [9]. Thus, it is likely that the anti-PIM antibody response developed by immunized cattle in this study was non-neutralizing. Thus, even robust humoral immune responses to this immunodominant antigen are not completely protective, as is the case with similar immunodominant proteins in other organisms [27], [28].

Finally, each vaccination with 10 GG cartridges utilized only ∼5 µg of DNA (0.5 µg/cartridge), and lower amounts of DNA per cartridge may be equally effective [28]. The dose-sparing nature of GG vaccination and relative stability of gene gun cartridges increase the utility of this platform for field immunization.

In conclusion, our data demonstrate that intradermal GG DNA immunization can elicit both humoral- and cell-mediated immunity to complex antigens of hemoparasitic pathogens of cattle. Codon-optimization of antigenic sequences for mammalian cell expression is critical to immune response development. Our system generated a Th1/IgG2-biased response, which is appropriate for the elicitation of protective immunity to intracellular hemoparasites. Our data also indicate that anti-PIM antibody responses are insufficient to prevent ECF upon *T. parva* challenge and that further work is needed to determine whether PIM is a viable CD8^+^ CTL target antigen in some cattle. With further optimization, GG DNA immunization is a promising delivery platform for next-generation *T. parva* vaccination, and further studies are underway to determine whether multivalent GG DNA immunization with known CD8^+^ CTL target antigens and sporozoite antibody targets will elicit protective immunity to *T. parva*.

## Competing interests

The authors have declared that no competing interests exist.

## Author contributions

**Conceptualization:** Lindsay M. Fry, Brad C. Stone, Reginaldo G. Bastos, Donald P. Knowles, and Sean C. Murphy

**Data curation:** Lindsay M. Fry, Reginaldo G. Bastos, Brad C. Stone

**Formal analysis:** Lindsay M. Fry, Reginaldo G. Bastos, Brad C. Stone, Laura B. Williams, Donald P. Knowles, Sean C. Murphy

**Funding acquisition:** Lindsay M. Fry, Donald P. Knowles

**Investigation:** Lindsay M. Fry, Reginaldo G. Bastos, Brad C. Stone, Laura B. Williams, Donald P. Knowles, Sean C. Murphy

**Methodology:** Lindsay M. Fry, Reginaldo G. Bastos, Brad C. Stone, Laura B. Williams, Donald P. Knowles, Sean C. Murphy

**Project administration:** Lindsay M. Fry, Donald P. Knowles, Sean C. Murphy

**Resources:** Lindsay M. Fry, Donald P. Knowles, Sean C. Murphy

**Supervision:** Lindsay M. Fry, Sean C. Murphy

**Validation:** Lindsay M. Fry, Brad C. Stone, Reginaldo G. Bastos, Sean C. Murphy

**Visualization:** Lindsay M. Fry

**Writing – original draft:** Lindsay M. Fry

**Writing – review & editing:** Lindsay M. Fry, Reginaldo G. Bastos, Brad C. Stone, Laura B. Williams, Donald P. Knowles, Sean C. Murphy

## Appendix A Supplementary material

Supplementary data 1 Alignment of codon-optimized (top) and native (bottom) PIM sequences, excluding introns.

Supplementary data 2 Codon optimization and in vitro expression of T. parva PIM. (A) Table showing the percentage of adenine (A), thymine (T), guanine (G), cytosine (C), and %GC content of native and codon-optimized (CO) PIM. (B) Immunoblot of HEK 293t cells or BAEC transiently transfected with a plasmid containing the native or c CO PIM sequence. Cells transfected with pMaxGFP (HEK-GFP or BAEC-GFP) were used a negative controls for immunoblots. (C) Densitometry analysis of immunoblots. Transfection/expression experiments were performed at least three times and densitometry results presented in panel C are representative of the immunoblot shown in panel B.

Supplementary data 3 Clinical response to T. parva challenge. For each parameter, black dots correspond to the mean value for PIM-immunized cattle, and grey dots to control cattle. Results in both groups are consistent with moderate to severe ECF, and no significant difference was observed in any clinical parameter. (A) Rectal temperature over time, normal < 39.4 °C. (B) Total white blood cell count over time (normal range: 2710–17,760 leukocytes/µL of blood). (C) Platelet count over time (normal range: 147,000–663,000 platelets/µL of blood). (D) T. parva parasite density over time as measured by T. parva p104 qPCR. All cattle developed qPCR-detectable infections between 10 and 13 days post-challenge, and no significant difference in parasite densities was observed between groups.

Supplementary data 4 PIM antigen immunohistochemistry, lymph node and lung from a representative (A) immunized and (B) control steer. Following challenge, there are abundant PIM-positive, schizont-infected lymphocytes within both the lung and lymph nodes of cattle from both groups. Scale bar: 50 μm.

## References

[1] Di Giulio G., Lynen G., Morzaria S., Oura C., Bishop R. (2008). Live immunization against East Coast Fever – Current Status. Trends Parastol.

[2] Fry L.M., Schneider D.A., Frevert C.W., Nelson D.D., Morrison W.I., Knowles D.P. (2016). East Coast Fever caused by *Theileria parva* is characterized by macrophage activation associated with vasculitis and respiratory failure. PLoS One.

[3] Norval R.A., Lawrence J.A., Young A.S., Perry B.D., Dolan T.T., Scott J. (1991). *Theileria parva*: influence of vector, parasite and host relationships on the epidemiology of theileriosis in southern Africa. Parasitology.

[4] McKeever D.J., Taracha E.L.N., Innes E.L., MacHugh N.D., Awino E., Goddeeris B.M. (1994). Adoptive transfer of immunity to *Theileria parva* in the CD8+ fraction of responding efferent lymph. Proc Natl Acad Sci.

[5] Morrison W.I., McKeever D.J. (2006). Current status of vaccine development against Theileria parasites. Parasitology.

[6] Morrison W.I., Goddeeris B.M., Teale A.J., Groocock C.M., Kemp S.J., Stagg D.A. (1987). Cytotoxic T cells elicited in cattle challenged with *Theileria parva* (Muguga): evidence for restriction by class I MHC determinants and parasite strain specificity. Parasite Immunol.

[7] Baldwin C., Iams K.P., Brown W.C., Grab D.J. (1992). *Theileria parva*: CD4+ helper and cytotoxic T-cell clones react with a schizont-derived antigen associated with the surface of *Theileria parva*-infected lymphocytes. Exp Parasitol.

[8] Kariuki D.P., Young A.S., Morzaria S.P., Lesan A.C., Mining S.K., Omwoyo P. (1995). *Theileria parva* carrier state in naturally infected and artificially immunised cattle. Trop Anim Health Prod.

[9] Nene V., Morrison W.I. (2016). Approaches to vaccination against *Theileria parva* and Theileria annulata. Parasite Immunol.

[10] Graham S.P., Pelle R., Honda Y., Mwangi D.M., Tonukari N., Yamage M. (2006). *Theileria parva* candidate vaccine antigens recognized by immune bovine cytotoxic T lymphocytes. Proc Natl Acad Sci.

[11] Nene V., Kiara H., Lacasta A., Pelle R., Svitek N., Steinaa L. (2016). The biology of *Theileria parva* and control of East Coast fever – Current status and future trends. Ticks Tick Borne Dis..

[12] Lacasta A., Mwalimu S., Kibwana E., Saya R., Awino E., Njoroge T. (2018). Immune parameters to p67C antigen adjuvanted with ISA206VG correlate with protection against East Coast fever. Vaccine.

[13] Norling M., Bishop R.P., Pelle R., Qi W., Henson S., Drabek E.F. (2015). The genomes of three stocks comprising the most widely utilized live sporozoite *Theileria parva* vaccine exhibit very different degrees and patterns of sequence divergence. BMC Genomics.

[14] Hemmink J.D., Sitt T., Pelle R., de Klerk-Lorist L.M., Shiels B., Toye P.G. (2018). Ancient diversity and geographical sub-structuring in African buffalo *Theileria parva* populations revealed through metagenetic analysis of antigen-encoding loci. Int J Parasitol.

[15] Morrison W.I., Connelley T., Hemmink J.D., MacHugh N.D. (2015). Understanding the basis of parasite strain-restricted immunity to *Theileria parva*. Annu Rev Anim Biosci.

[16] Pelle R., Graham S.P., Njahira M.N., Osaso J., Saya R.M., Odongo D.O. (2011). Two *Theileria parva* CD8 T cell antigen genes are more variable in buffalo than cattle parasites, but differ in pattern of sequence diversity. PLoS One.

[17] MacHugh N.D., Connelley T., Graham S.P., Pelle R., Formisano P., Taracha E.L. (2009). CD8+ T-cell responses to *Theileria parva* are preferentially directed to a single dominant antigen: implications for parasite strain-specific immunity. Eur J Immunol.

[18] Codner G., Stear M., Reeve R., Matthews L., Ellis S. (2011). Selective forces shaping diversity in the class I region of the major histocompatibility complex in dairy cattle. Anim Genet.

[19] Codner G.F., Birch J., Hammond J.A., Ellis S.A. (2012). Constraints on haplotype structure and variable gene frequencies suggest a functional hierarchy within cattle MHC class I. Immunogenetics.

[20] Glass E.J. (2004). Genetic variation and responses to vaccines. Anim Health Res Rev.

[21] Ellis S.A., Hammond J.A. (2014). The functional significance of cattle major histocompatibility complex class I genetic diversity. Annu Rev Anim Biosci.

[22] Fuller D.H., Loudon P., Schmaljohn C. (2006). Preclinical and clinical progress of particle-mediated DNA vaccines for infectious diseases. Methods.

[23] Engelke L., Winter G., Hook S., Engert J. (2015). Recent insights into cutaneous immunization: how to vaccinate via the skin. Vaccine.

[24] Yager E.J., Stagnar C., Gopalakrishnan R., Fuller J.T., Fuller D.H. (2013). Optimizing particle-mediated epidermal delivery of an influenza DNA vaccine in ferrets. Methods Mol Biol.

[25] Chase C.C.L.D., Scanlon C., Garcia Roberto, Milward Frank, Nation Tiffany (2008). Needle-free injection technology in swine: progress toward vaccine efficacy and pork quality. J Swine Health Prod.

[26] Pertmer T.M., Eisenbraun M.D., McCabe D., Prayaga S.K., Fuller D.H., Haynes J.R. (1995). Gene gun-based nucleic acid immunization: elicitation of humoral and cytotoxic T lymphocyte responses following epidermal delivery of nanogram quantities of DNA. Vaccine.

[27] Murphy S.C., Kas A., Stone B.C., Bevan M.J. (2013). A T-cell response to a liver-stage Plasmodium antigen is not boosted by repeated sporozoite immunizations. Proc Natl Acad Sci USA.

[28] Stone B.C., Kas A., Billman Z.P., Fuller D.H., Fuller J.T., Shendure J. (2016). Complex minigene library vaccination for discovery of pre-erythrocytic plasmodium t cell antigens. PLoS One.

[29] Toye P., Gobright E., Nyanjui J., Nene V., Bishop R. (1995). Structure and sequence variation of the genes encoding the polymorphic, immunodominant molecule (PIM), an antigen of *Theileria parva* recognized by inhibitory monoclonal antibodies. Mol Biochem Parasitol.

[30] Toye P., Nyanjui J., Goddeeris B., Musoke A.J. (1996). Identification of neutralization and diagnostic epitopes on PIM, the polymorphic immunodominant molecule of *Theileria parva*. Infect Immun.

[31] Toye P., Musoke A., Naessens J. (2014). Role of the polymorphic immunodominant molecule in entry of *Theileria parva* sporozoites into bovine lymphocytes. Infect Immun.

[32] Toye P.G., Goddeeris B.M., Iams K., Musoke A.J., Morrison W.I. (1991). Characterization of a polymorphic immunodominant molecule in sporozoites and schizonts of *Theileria parva*. Parasite Immunol.

[33] Katende J., Morzaria S., Toye P., Skilton R., Nene V., Nkonge C. (1998). An enzyme-linked immunosorbent assay for detection of *Theileria parva* antibodies in cattle using a recombinant polymorphic immunodominant molecule. Parasitol Res.

[34] Benedictus L., Thomas A.J., Jorritsma R., Davies C.J., Koets A.P. (2012). Two-way calf to dam major histocompatibility class I compatibility increases risk for retained placenta in cattle. Am J Reprod Immunol.

[35] Gardner M.J., Bishop R., Shah T., de Villiers E.P., Carlton J.M., Hall N. (2005). Genome sequence of *Theileria parva*, a bovine pathogen that transforms lymphocytes. Science.

[36] Wise L.N., Kappmeyer L.S., Silva M.G., White S.N., Grause J.F., Knowles D.P. (2018). Verification of post-chemotherapeutic clearance of Theileria equi through concordance of nested PCR and immunoblot. Ticks Tick Borne Dis.

[37] Goddeeris B.M., Morrison W.I. (1988). Techniques for the generation, cloning, and characterization of bovine cytotoxic T cells specific for the protozoan *Theileria parva*. J Tissue Cult Methods.

[38] Odongo D.O., Ueti M.W., Mwaura S.N., Knowles D.P., Bishop R.P., Scoles G.A. (2009). Quantification of *Theileria parva* in Rhipicephalus appendiculatus (Acari: Ixodidae) confirms differences in infection between selected tick strains. J Med Entomol.

[39] Mukhebi A.W., Norval R.A.I., Perry B.D., Young A.S. (1992). The economic impact of Theileriosis and its control in Africa. The Epidemiology of Theileriosis in Africa.

[40] Loehr B.I., Willson P., Babiuk L.A., van Drunen Littel-van den H. (2000). Gene gun-mediated DNA immunization primes development of mucosal immunity against bovine herpesvirus 1 in cattle. J Virol.

[41] Braun R.P., Babiuk L.A., Loehr B.I., van Drunen Littel-van den H. (1999). Particle-mediated DNA immunization of cattle confers long-lasting immunity against bovine herpesvirus-1. Virology.

[42] van Drunen Littel-van den H., Braun R.P., Lewis P.J., Karvonen B.C., Baca-Estrada M.E., Snider M. (1998). Intradermal immunization with a bovine herpesvirus-1 DNA vaccine induces protective immunity in cattle. J Gen Virol.

[43] McGuire T.C., Musoke A.J., Kurtti T. (1979). Functional properties of bovine IgG1 and IgG2: interaction with complement, macrophages, neutrophils and skin. Immunology.

[44] Estes D.M., Brown W.C. (2002). Type 1 and type 2 responses in regulation of Ig isotype expression in cattle. Vet Immunol Immunopathol.

[45] Estes D.M., Closser N.M., Allen G.K. (1994). IFN-gamma stimulates IgG2 production from bovine B cells costimulated with anti-mu and mitogen. Cell Immunol.

[46] Estes D.M., Hirano A., Heussler V.T., Dobbelaere D.A., Brown W.C. (1995). Expression and biological activities of bovine interleukin 4: effects of recombinant bovine interleukin 4 on T cell proliferation and B cell differentiation and proliferation in vitro. Cell Immunol.

[47] Estes D.M., Tuo W., Brown W.C., Goin J. (1998). Effects of type I/type II interferons and transforming growth factor-beta on B-cell differentiation and proliferation. Definition of costimulation and cytokine requirements for immunoglobulin synthesis and expression. Immunology.

[48] Trigona W.L., Hirano A., Brown W.C., Estes D.M. (1999). Immunoregulatory roles of interleukin-13 in cattle. J Interferon Cytokine Res.

[49] Sugimoto C., Mutharia L.M., Brown W.C., Pearson T.W., Dolan T.T., Conrad P.A. (1992). Analysis of *Theileria parva* immunodominant schizont surface antigen by two-dimensional polyacrylamide gel electrophoresis and immunoblotting. Parasitol Res.

[50] Graham S.P., Pelle R., Yamage M., Mwangi D.M., Honda Y., Mwakubambanya R.S. (2008). Characterization of the fine specificity of bovine CD8 T-cell responses to define antigens from the protozoan parasite *Theileria parva*. Infect Immun.

[51] Morrison W.I. (2015). The aetiology, pathogenesis and control of theileriosis in domestic animals. Rev Sci Tech.

