# Supplementary material for: Gene gun DNA immunization of cattle induces humoral and CD4 T-cell-mediated immune responses against the Theileria parva polymorphic immunodominant molecule
Source: Vaccine. 2019 Mar 14;37(12):1546–53. doi: 10.1016/j.vaccine.2019.02.009 (PMC6411927; doi:10.1016/j.vaccine.2019.02.009)
Supplement: Supplementary data 1 — Alignment of codon-optimized (top) and native (bottom) PIM sequences, excluding introns. [file mmc1.pdf]

Supplementary Fig. 1

Alignment of codon-optimized and native PIM sequences

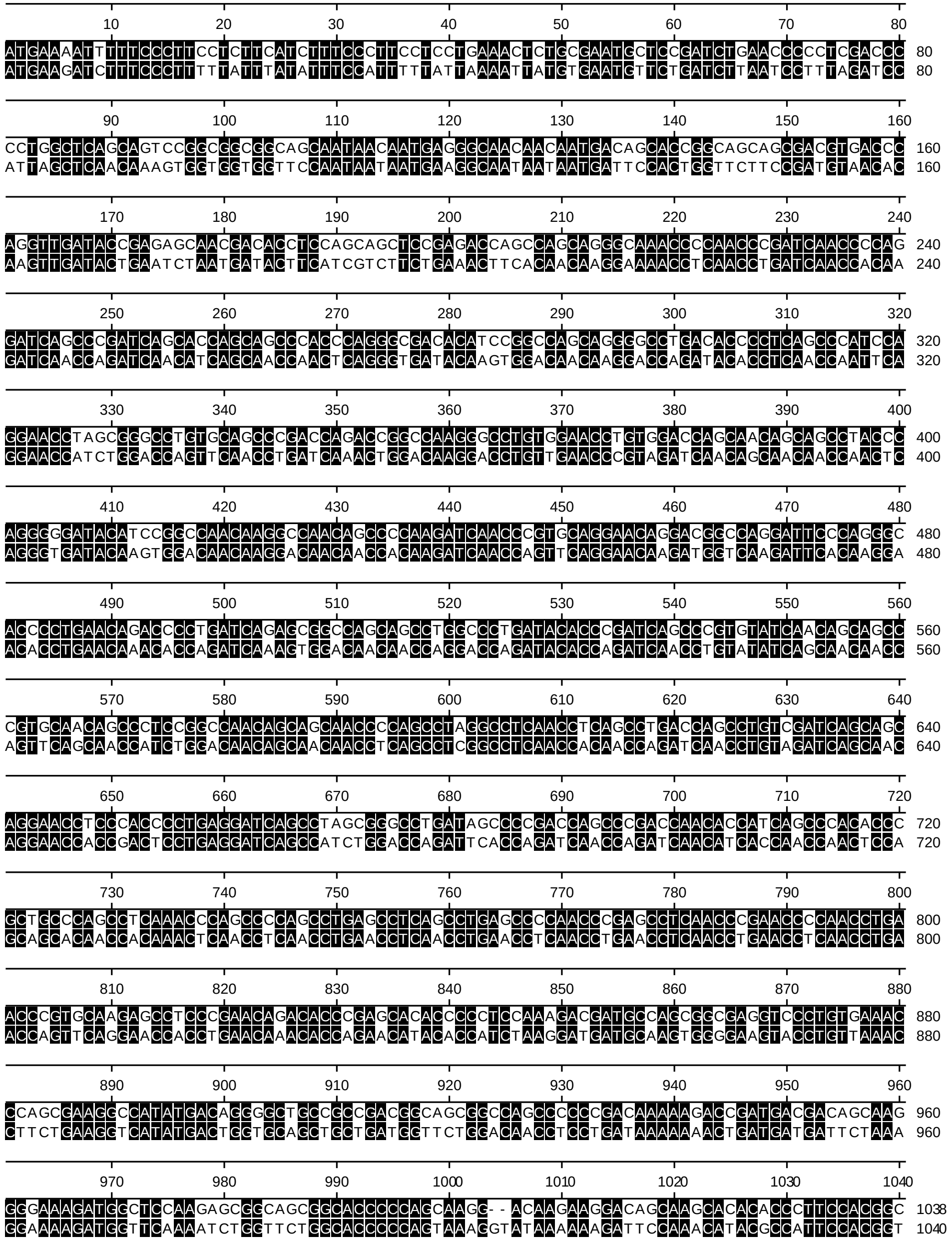

Supplementary Fig. 1

Alignment of native and codon-optimized PIM sequences (continued)

```
         1050      1060      1070      1080      1090      1100      1110      1120
TGCGGGCAGTTCTTTACCAATACCCACAAAGTGACCGTGATCTTTCACTGGTGGCTGTGCGAAAAGCCC-TGGCAGTATG 1117
TGTGGGCAATTCTTCACAAATACACATAAAGTCACCGTTATTTTCCATTGGTGGTTGTGTGAAAAGGTCATGGCAATACG 1120

         1130      1140      1150      1160      1170      1180      1190      1200
CCCTGACCCTGCTGACCCTCTTCGGCTTTGCTCTGCTGAGCCCTTGCCTGAAGGCCTACAGGGAGGTGCTGAGGGCCAAG 1197
CTCTAACATTGTTGACTTTGTTTGGTTTTGCACTTTTGAGTCCATGTTTAAAGGCCTATAGAGAGGTTCTCAGAGCAAAG 1200

         1210      1220      1230      1240      1250      1260      1270      1280
GCCGTGAGGAGCTTCATCTTCGACTGCCTCCTGACCCACCTGTTCCTGTTCCTCATTGCCCTCTGCGCCTATGCCCTGGA 1277
GCGGTAAGAAGTTTCATTTTCGACTGTCTTTTGACACATCTCTTCCTCTTTTTGATTGCACTTTGTGCCTACGCATTGGA 1280

         1290      1300      1310      1320      1330      1340      1350      1360
CTTCCTGCTCATGCTGGTGGTGATGACCTTTAACGTGGGGGTGTTTTTCGCCGTGATCCTCGGCTATAGCGTGGGCTACG 1357
CTTTTTGCTCATGCTTGTTGTAATGACCTTCAATGTTGGTGTATTCTTCGCCGTTATCTTGGGCTATTCCGTAGGATACG 1360

         1370      1380      1390      1400      1410      1420      1430      1440
TGCTGAGCAGCCTGGCTTATTCCACCCTCCGGACCCAGCCTAACCGGTCCAACTCCTTTAGCCGGATCAATGAGGACTGC 1437
TACTATCCTCGTTAGCCTATTCAACGTTACGTACTCAACCCAATAGAAGCAACTCTTTTTCTCGCATTAACGAAGATTGT 1440

TGCTGA 1443
TGTTAA 1446
```
