# Supplementary figures and images for: Gene gun DNA immunization of cattle induces humoral and CD4 T-cell-mediated immune responses against the Theileria parva polymorphic immunodominant molecule
Source: Vaccine. 2019 Mar 14;37(12):1546–53. doi: 10.1016/j.vaccine.2019.02.009 (PMC6411927; doi:10.1016/j.vaccine.2019.02.009)

Supplementary Fig. 2

A

| PIM Construct   | %A   | %T   | %C   | %G   | %GC Content |
|-----------------|------|------|------|------|-------------|
| Native          | 32.6 | 25.1 | 24.8 | 17.3 | 42.3        |
| Codon Optimized | 23.2 | 14.7 | 37.1 | 24.8 | 62.0        |

B

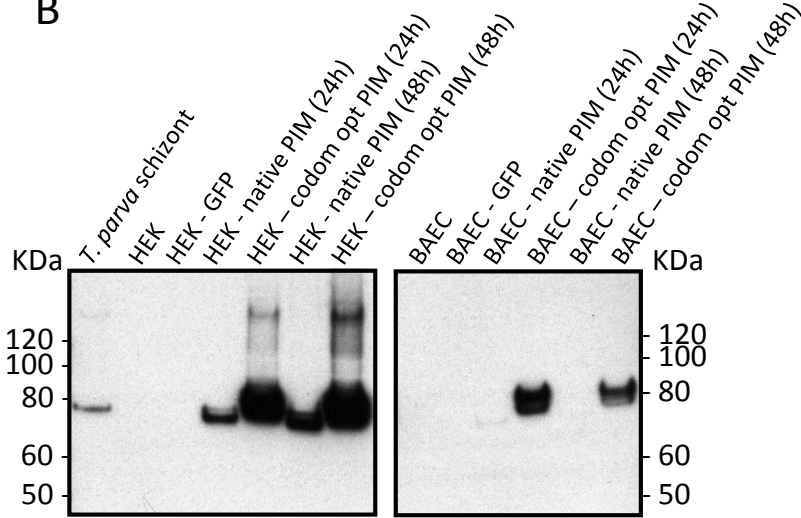

C

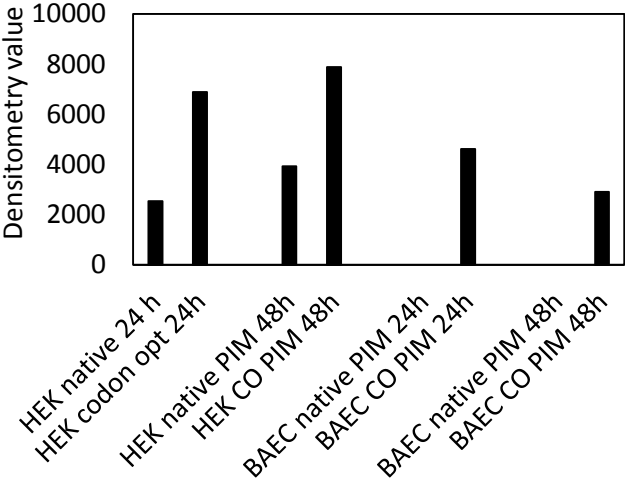

Supplement: Supplementary data 2 — Codon optimization and in vitro expression of T. parva PIM. (A) Table showing the percentage of adenine (A), thymine (T), guanine (G), cytosine (C), and %GC content of native and codon-optimized (CO) PIM. (B) Immunoblot of HEK 293t cells or BAEC transiently transfected with a plasmid containing the native or c CO PIM sequence. Cells transfected with pMaxGFP (HEK-GFP or BAEC-GFP) were used a negative controls for immunoblots. (C) Densitometry analysis of immunoblots. Transfection/expression experiments were performed at least three times and densitometry results presented in panel C are representative of the immunoblot shown in panel B. [file mmc2.pdf]

Supplementary Fig. 3

A

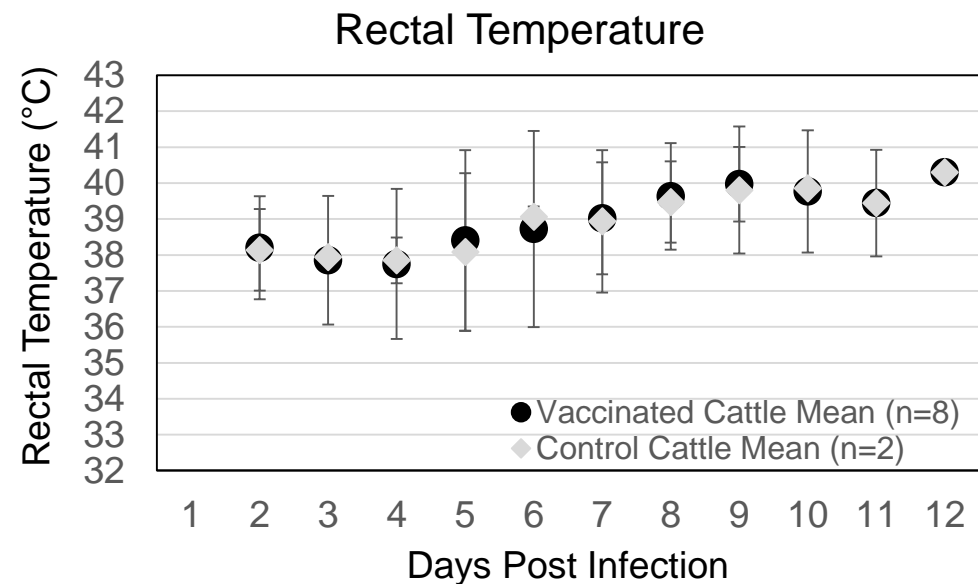

B

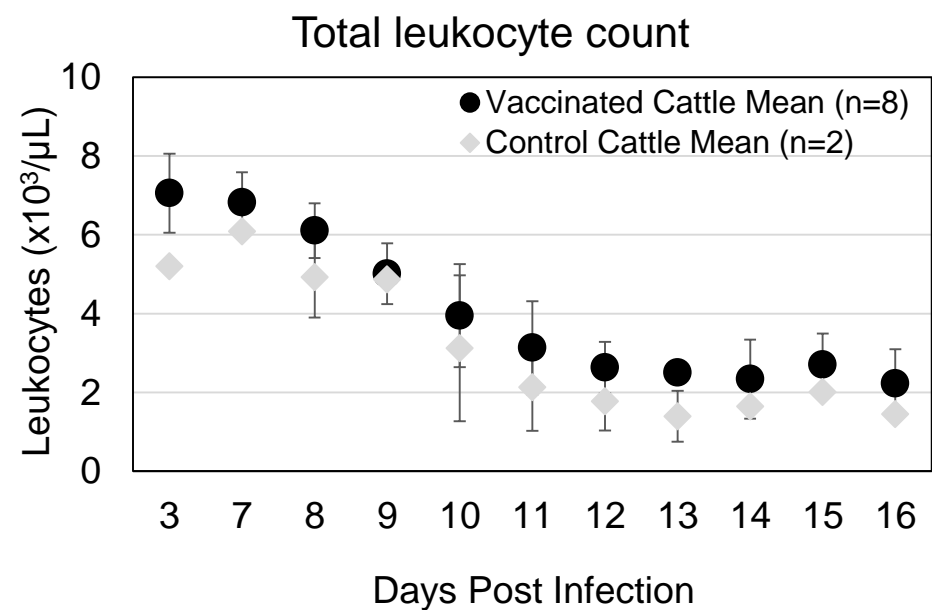

C

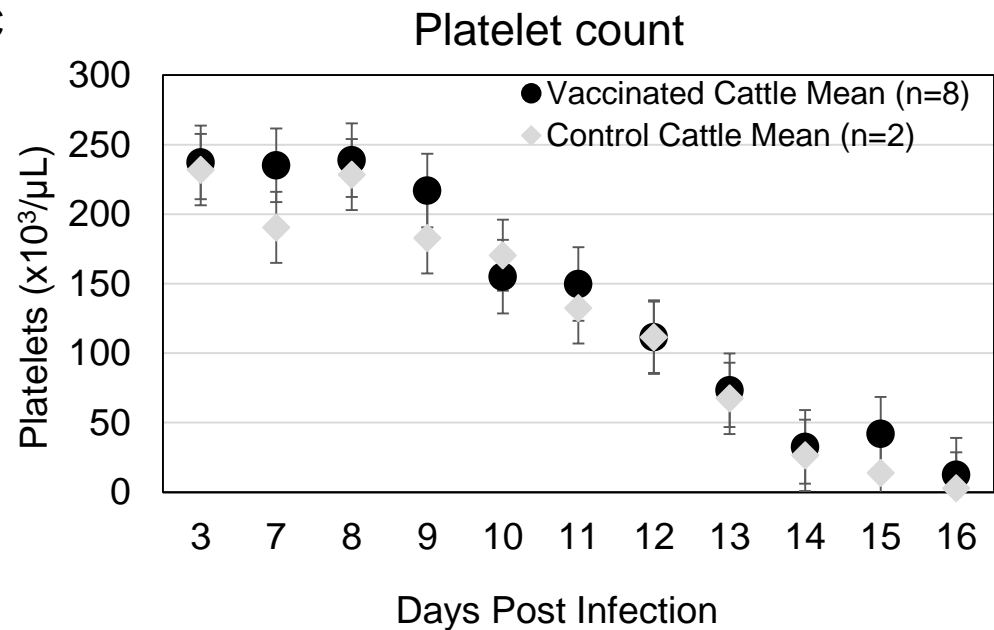

D

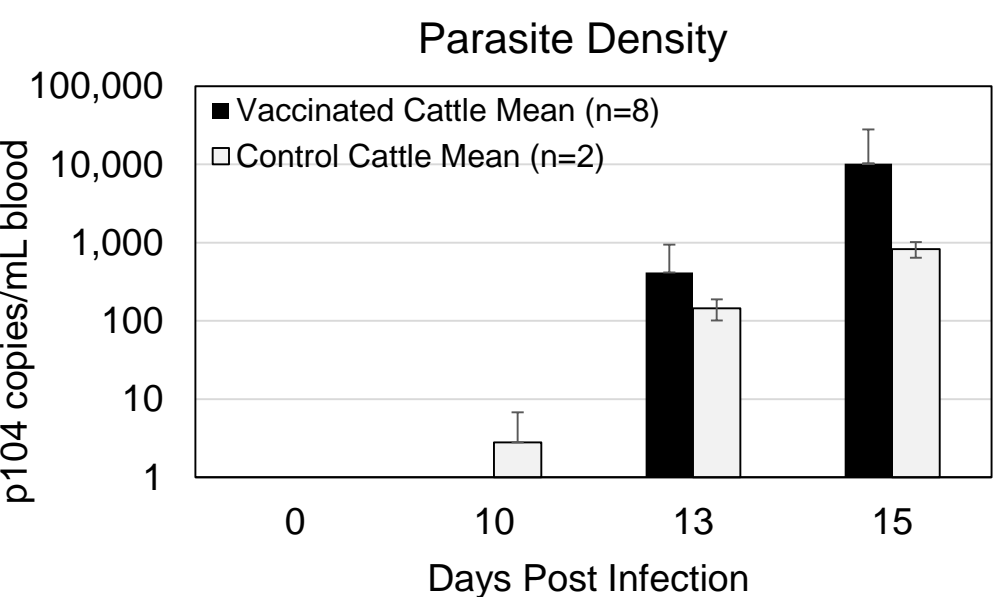

Supplement: Supplementary data 3 — Clinical response to T. parva challenge. For each parameter, black dots correspond to the mean value for PIM-immunized cattle, and grey dots to control cattle. Results in both groups are consistent with moderate to severe ECF, and no significant difference was observed in any clinical parameter. (A) Rectal temperature over time, normal < 39.4 °C. (B) Total white blood cell count over time (normal range: 2710–17,760 leukocytes/µL of blood). (C) Platelet count over time (normal range: 147,000–663,000 platelets/µL of blood). (D) T. parva parasite density over time as measured by T. parva p104 qPCR. All cattle developed qPCR-detectable infections between 10 and 13 days post-challenge, and no significant difference in parasite densities was observed between groups. [file mmc3.pdf]

Supplementary Fig. 4

Lymph node

Lung

A

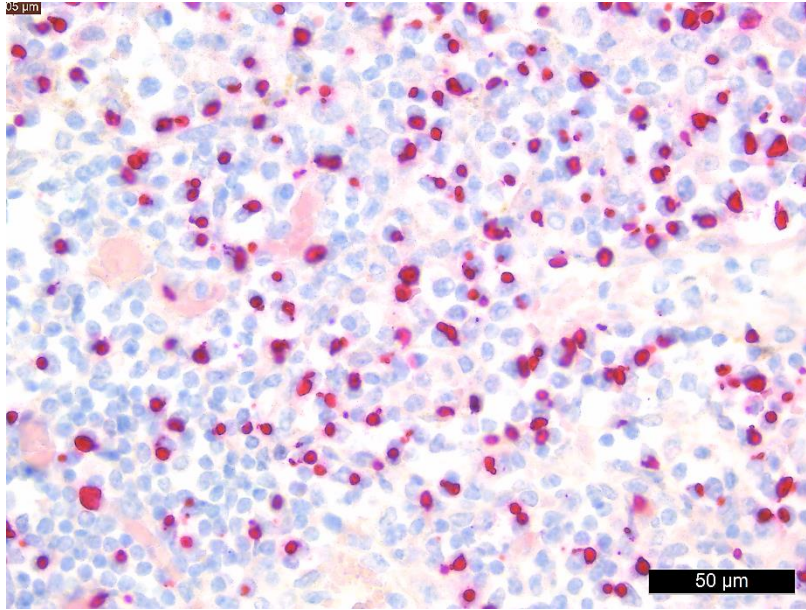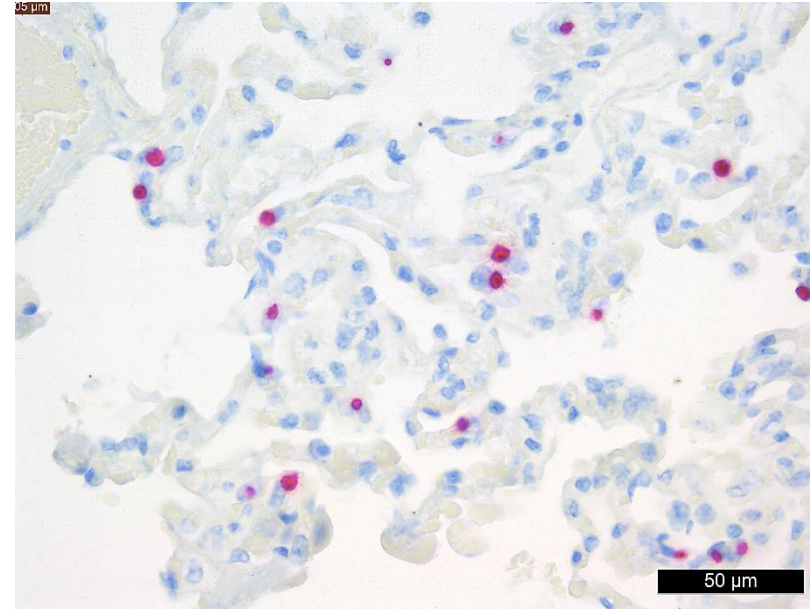

B

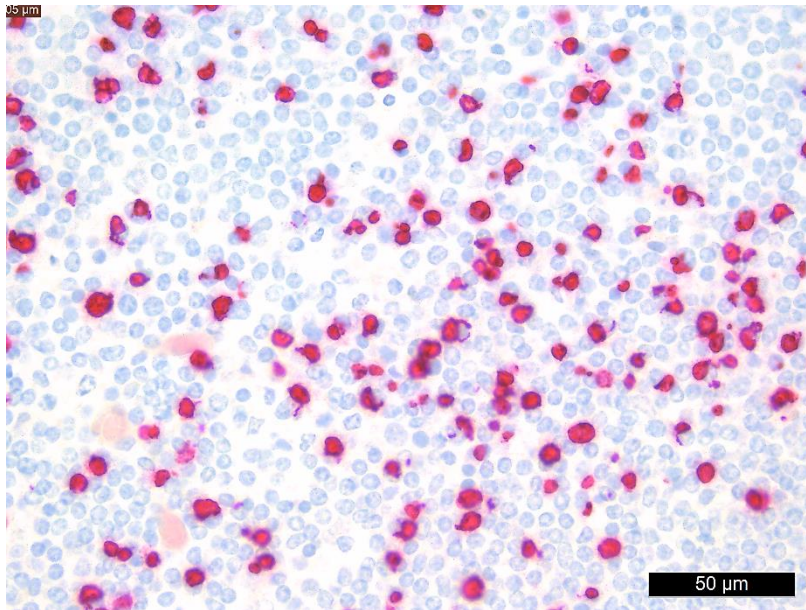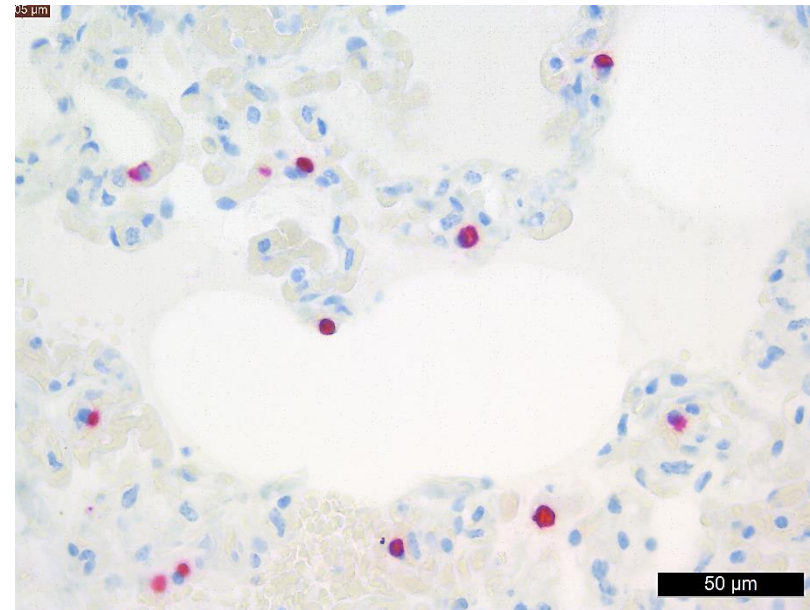

Supplement: Supplementary data 4 — PIM antigen immunohistochemistry, lymph node and lung from a representative (A) immunized and (B) control steer. Following challenge, there are abundant PIM-positive, schizont-infected lymphocytes within both the lung and lymph nodes of cattle from both groups. Scale bar: 50 μm. [file mmc4.pdf]
